# Designing evaluation infrastructure for psychedelic harm reduction at events: a methodological framework with formative mixed-methods field application

**DOI:** 10.3389/fpubh.2026.1927839

**Published:** 2026-09-29

**Authors:** Nima Veiseh, Francesco Kment, Victor Acero, Heather Jackson, Ruth Fisher, Case Newsom, Jess Aumick, Armando León, Ishaq Ali, Jessa Hurst, Sydney N. Lee, Alex Utay, Laila Murfin

**Affiliations:** 1 Zendo Project, Grass Valley, CA, United States; 2 Walden University, Minneapolis, MN, United States; 3 Mindful Conversations Psychotherapy, Oshawa, ON, Canada; 4 Center for Psychedelic Drug Research and Education, The Ohio State University, Columbus, OH, United States; 5 Unlimited Sciences, Colorado Springs, CO, United States; 6 CannDynamics, St. Petersburg, FL, United States; 7 Stadium Medical, Denver, CO, United States; 8 San Mateo Medical Center, San Mateo, CA, United States; 9 UC Berkeley Cannabis Research Center, Berkeley, CA, United States; 10 Teachers College, Columbia University, New York, NY, United States; 11 Columbia University, New York, NY, United States

**Keywords:** crisis response, event medicine, harm reduction, mass-gathering events, peer support, psychedelics, public health infrastructure, public policy

## Abstract

Psychedelic and other psychoactive substance use is increasing in non-clinical environments, including music festivals, concerts, and other large-scale gatherings. As psychedelic use expands beyond clinical settings, policymakers, healthcare systems, event operators, and researchers require evidence-informed frameworks for understanding and responding to psychoactive-related distress in naturalistic environments. Despite the growing presence of psychedelic harm reduction services, few studies have established reproducible methods for documenting their operation, coordination with event-health services, or role within triage and escalation pathways in naturalistic settings. This methodological paper proposes an evaluation framework for psychedelic harm reduction services and reports a formative mixed-methods field application involving the Zendo Project at two large-scale events. Nineteen pre-event surveys, five retained post-event surveys, and two matched pre/post records were obtained from medical personnel. Quantitative responses were summarized descriptively, while open-ended responses were used formatively to contextualize and refine the proposed measurement domains. The framework positions psychedelic peer support as a complementary intermediate pathway for medically stable but psychologically distressed individuals, operating alongside rather than in place of medical and public-safety services. The field application identified interpretable measurement domains concerning psychological-distress burden, preparedness, workload, safety, interprofessional coordination, escalation, and attendee disposition. Four respondents completed several principal post-event items and reported favorable perceptions of Zendo's operational role. These responses represent staff perceptions and counterfactual estimates rather than independently verified effects. Beyond evaluating a single intervention, this study offers an initial framework for measuring and comparing non-clinical psychedelic support models and for informing future research, policy development, and event-health infrastructure as psychedelic use continues to expand beyond clinical settings. Additionally, by translating field-based psychedelic support into measurable operational, public health, and economic outcomes, the proposed methodology offers a foundation for the future evaluation, comparison, and development of products, services, and care infrastructures designed to support altered-state experiences in non-clinical environments.

## Introduction

The prevalence of psychedelic substance use has increased in recent years, particularly in non-clinical environments such as music festivals, concerts, and other large-scale public gatherings (1, 2). While much contemporary research has focused on the therapeutic potential of psychedelics in controlled clinical settings, the majority of use continues to occur in naturalistic contexts, where environmental, social, and physiological variables are less regulated (3, 4). These settings introduce distinct public health considerations, as risk is shaped not only by pharmacological effects but also by factors such as crowd, density, heat exposure, weather, sleep deprivation, and polysubstance use (5, 6).

Although serious medical complications associated with classic psychedelics are relatively rare compared to overall rates of use, acute adverse experiences–often characterized by anxiety, panic, paranoia, or confusion–are well documented (7, 8). Importantly, these presentations are frequently psychological rather than physiological in nature and may not require medical intervention when appropriately supported (7). However, in large-scale event environments, individuals experiencing such distress may often default into emergency response pathways involving emergency medical services (EMS), venue security, or law enforcement, systems that are not primarily designed to address psychologically driven altered states (6, 9).

Mass-gathering health literature has increasingly emphasized the importance of integrated and context-sensitive care models to manage the diverse range of presentations encountered in these environments (9, 10). Psychosocial stressors, environmental conditions, and group dynamics have all been shown to influence both the frequency and nature of patient presentations, as well as the burden placed on emergency services (6). Meanwhile, research on mental health crisis response suggests that health-oriented or peer-based models may offer useful alternatives to exclusively law-enforcement-based responses in some crisis contexts (11). Together, these findings point to a gap in current event response systems: the absence of a structured, intermediate layer of care designed specifically for non-medical, psychologically driven distress.

The Zendo Project represents one of the most established field-based applications of psychedelic harm reduction principles in large-scale event settings. Developed as a peer-support model, the Zendo Project provides non-judgmental, psychologically informed care for individuals undergoing difficult psychedelic experiences, with an emphasis on de-escalation, environmental safety, and supportive presence. This may also be commonly referred to as a sanctuary space. Importantly, the model operates in coordination with existing emergency and medical services, maintaining clear criteria for escalation when medical or safety risks exceed the scope of peer support. Therefore, it may function as a complementary layer within event response systems, addressing a category of distress that may not be optimally managed by traditional emergency frameworks.

Although psychedelic harm reduction services have become increasingly visible in large-scale event settings, their operational role remains underexamined in the empirical literature. Existing work has primarily discussed these models conceptually or descriptively, with limited research evaluating how they function in real-world environments, how support encounters are documented and how harm reduction teams coordinate emergency medical services. Addressing this gap is important as psychedelic use continues outside clinical contexts and event systems seek evidence-informed approaches to psychological distress, triage, and escalation.

The present study has two related aims. First, it proposes a methodological framework for evaluating psychedelic harm reduction services within large-scale event-health systems. Second, it reports a formative field application used to assess survey feasibility, identify practical data-collection challenges, and refine candidate measurement domains. The two-event application was not designed to determine effectiveness or quantify reductions in workload, escalation, transport, or resource use. Welfare-economic and ASCI-related components are presented as prospective extensions of the framework that require independent empirical testing.

## Literature review

### The two-pillar reality and the missing third

When crises occur at large public events, two infrastructures respond. Emergency medical services handle physiological emergencies, and security and/or law enforcement handles incident control. Neither system is specifically designed to provide sustained psychological support for medically stable individuals experiencing acute psychological or altered-state distress, a category broader than psychedelic crises that also includes anxiety, dissociation, panic, and emotional crises unrelated to any substance (12). Recreational psychedelic use among nightclub and festival attendees has risen sharply, a sentinel signal of wider drug trends (13), and difficult experiences, marked by near-ubiquitous fear and frequent confusion, are a common feature of that use (14). The two existing pillars are poorly matched to these presentations, and people in distress often fall between them, turned away by medics who see no medical problem and by security and police who see no crime (15). Help-seeking is further suppressed by fear of cost and legal exposure, so guests avoid both pillars even when they need them (16, 17). The event-safety literature already anticipates the missing element, naming a distinct “festival health” function alongside policing and medical response and warning that siloed planning produces gaps (18, 19). This literature suggests a potential role for a complementary event-health pathway consisting of non-clinical harm reduction operating alongside EMS, security, and law-enforcement services (Figure 1).

**Figure 1:**
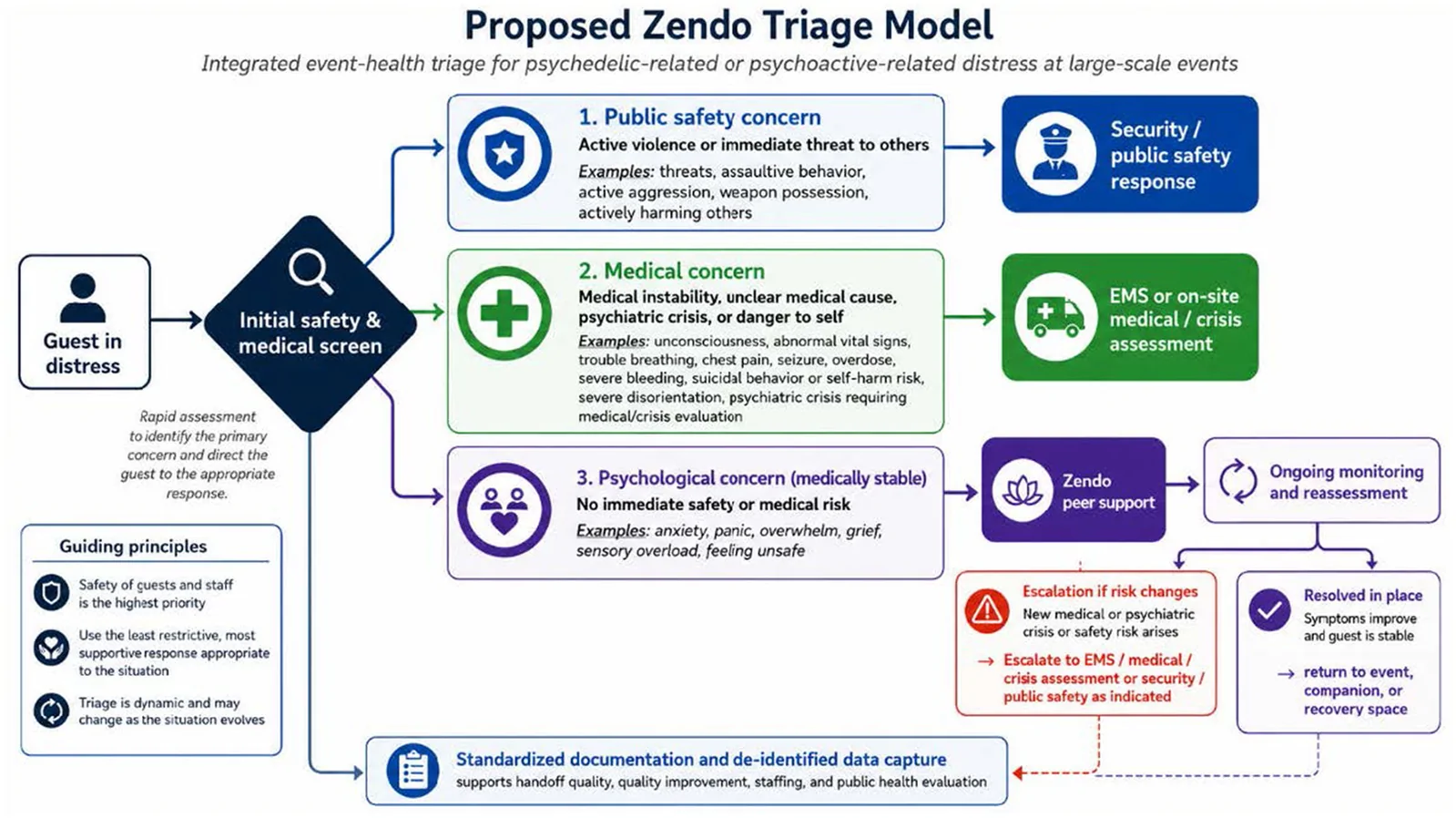
Proposed Zendo Project triage model for psychedelic or psychoactive distress at large-scale events. The model depicts initial safety and medical screening, routing to public safety, EMS/on-site medical care, or Zendo peer support, dynamic reassessment, and standardized documentation for quality improvement and public health evaluation.

### The burden on existing systems

Large events generate substantial medical workloads, and demand from altered-state presentations is rising: hallucinogen-associated emergency-department visits and hospitalizations in California climbed by more than half between 2016 and 2022, even as alcohol-related visits fell (20), and single events can produce sharp local surges in drug-related ambulance and emergency-department contacts (21). Each avoidable transport or emergency department visit may consume capacity intended for higher-acuity physiological emergencies and contribute to broader system strain. Where on-site harm reduction operates in parallel with medical services, preliminary case-mix data suggest that some low-acuity psychological or substance-related presentations may be managed without transport, preserving medical resources for cases requiring clinical intervention (22).

### Existing peer-support models and preliminary evidence

For this population, psychological care rarely arrives through mainstream healthcare systems. It comes instead through non-profit, pre-profit, and community organizations. The field-based harm-reduction traditions of Kosmicare, Zendo Project, and the broader network of peer organizations provide psychological support (23, 24). Peer support is the mechanism that pathway runs on, and it provides what people in difficulty most often say helps them [feeling heard, acceptance, and shared experience; (25)]. The Zendo Project has distilled these into four operational principles (Safe Space, Sitting Not Guiding, Talking Through Not Down, and Difficult Is Not the Same as Bad), refined over more than a decade of field practice (26). The model is teachable, producing measurable gains in non-clinicians' capacity to support others in distress (27). Its outcomes bear directly on the concerns that motivate this paper: a psychedelic peer-support helpline de-escalated most callers and diverted a meaningful share who would otherwise have called 911 or gone to an emergency room, easing the burden on emergency and medical services (28), and the one evaluated festival safe-space service significantly resolved acute psychedelic and emotional crises (29). On-site case-mix data are likewise consistent with harm reduction drawing low-acuity presentations away from EMS (22). The contrast with the clinical literature is instructive: the substances present at these events are supported by an evidence base reaching randomized controlled trials (30, 31), while the field practice that meets them rests on scoping reviews, ethnography, and descriptive case reports: a literature that establishes the practice exists, but not how well it works or how to measure it.

### Research gap and framework contribution

That asymmetry is the gap. The instruments that would let the field measure itself are partial. The most-cited instrument for difficult psychedelic experiences, the Challenging Experience Questionnaire, was constructed and validated through retrospective internet surveys of past experiences, for use in research (32). No comparable instrument exists for assessing distress in real time during a field encounter. Event-medicine reporting frameworks do not capture the harm-reduction pillar, which goes effectively unmeasured (33). A recent scoping review found only four evaluated interventions among twenty-seven studies (34). The limitation is acknowledged within the team, where the conceptual framework and the field data-collection application were developed because no validated instrument existed. The literature thus points to a clear conclusion: a real gap, practice that already fills it, and an evidence base too partial to integrate it as policy. The present study responds to this gap by proposing an integrated and testable measurement framework. The framework encompasses operational documentation, staff and stakeholder perspectives, objective event-health outcomes, resource-use measures, and prospective relational constructs. The two-event field application addresses only a subset of these components and is used formatively to refine survey domains and implementation procedures.

### Introduction to policy constraints

The legal environment around drug policy significantly influences the feasibility of research and program development within community-based psychedelic harm reduction frameworks. The federal RAVE Act (the Illicit Drug Anti-Proliferation Act of 2003), the foundational policy constraint in the United States, establishes severe gray-market liability concerns for event organizers. Mohr (35) and Ratushniak ACH (36) both emphasize that fear of prosecution under the RAVE Act discourages formal tool creation, open communication, and the visible deployment of harm reduction services and programs at music festivals and public gatherings, inadvertently compounding real-world risks while ignoring the reality of substance use at certain events. Mohr (35) points out that although the RAVE Act has not been used for criminal enforcement, it creates enough liability for event organizers that they cannot get insurance for the event, effectively precluding on-site harm reduction services altogether.

Pilecki et al. (37) assert that over 32 million people in the USA have tried psychedelics in their lifetime; that “members of the public seek to use psychedelics for therapeutic purposes but are unsure of how to do so in a way that is physically, psychologically, and legally safe.” This leads many people to accessing psychedelics in underground or non-clinical environments such as music festivals (36). Ratushniak further observes that music festivals themselves can be over-stimulating environments and coupled with psychedelics, can be a recipe for an acute mental health reaction therefore, it is important for organizers to plan accordingly. Unfortunately, like in the USA, in Canada, “liability concerns are often cited as the reason why a festival cannot provide drug testing kits or services” according to Ratushniak.

Consequently, legal and ethical anxieties create a chilling effect on researchers attempting to document and evaluate peer-support practices in gray-market environments. In order to move past these structural barriers, psychedelic harm-reduction frameworks need to transition toward an integrated public health approach that balances ethical responsibilities and legal compliance. In the research that does exist, scholars advocate for a formalized public-health-based framework for psychedelic regulation that emphasizes safety and harm minimization over criminalization (37–39).

### Access frameworks and regulatory precedent

Existing international harm-reduction or psychedelic access frameworks offer potential policy analogs when designing viable regulatory models in the United States. For example, Canada has provided access points through its Drug Exceptional Access Programs (DEAPs), roadmaps for psychedelic pharmacy integration, and evaluations of patient experiences with compassionate psilocybin access (40–42). Furthermore, as mainstream acceptance of psychedelic therapy continues to grow, research urges practitioners to begin developing and integrating safety, clinical, ethical, and other relevant protocols around psychedelics in fields such as therapy, psychiatry, and pharmacology (40–42).

These frameworks highlight the ongoing tensions between formal clinical regulation and increasing demand for real-world accessibility. Without comprehensive federal guidelines and a growing trend of local decriminalization movements (43), unregulated gray-market commercial spaces often emerge, such as unlicensed dispensaries and unsubstantiated advertising, which introduces further public safety and regulatory complications (44, 45). As decriminalized and newly regulated access increases, so does use in non-clinical settings.

### Veterans as a case study

An important driver of psychedelic policy and institutional acceptance is the expanding body of research around military veterans. Existing studies tracking the epidemiology of psychedelic use among U.S. and Canadian veterans indicate that treatment-resistant conditions, such as PTSD and traumatic brain injury (TBI), frequently motivate individuals to seek alternative care pathways (46–48).

Despite persistent institutional barriers, veteran-led advocacy and research have successfully functioned as a political wedge, accelerating legislative reforms and legitimizing conversations around broader medical and compassionate access (49). In 2025, Governor Abbott of Texas signed Senate Bill 2308, which authorizes state matching funds for ibogaine research as a potential treatment for neurological and mental health conditions, particularly among veterans (50). In April 2026, President Trump issued Executive Order 14,401, directing federal agencies to accelerate research and appropriate approval pathways for psychedelic drugs and to expedite rescheduling review for qualifying products (51).

### Cannabis as an analog and localized policy

The historical trajectory of cannabis policy provides a point of comparison for understanding contemporary psychedelic harm reduction policy development. Research demonstrates that institutional stigma and systemic deficits in healthcare provider education significantly hinder the integration of cannabis as a legitimate harm reduction strategy for substance use disorders (52–54).

Similar stigma constrained the field of psychedelic research and program development and events. Furthermore, a critical gap exists in current literature regarding localized U.S. state frameworks. While state-level initiatives like Oregon's Measure 109, Colorado's Proposition 122 (Natural Medicine Health Act), and New Mexico's HB7 are actively shaping their local landscape, peer-reviewed literature documenting their implementation and outcomes in non-clinical environments such as music festivals remain limited. Bridging this gap is essential for building standardized, legally viable toolkits capable of measuring harm reduction impact across real-world settings.

## Methodological framework development and formative field application

This paper presents a methodological framework with an embedded descriptive mixed-methods feasibility component. The framework specifies domains and data sources for the prospective evaluation of psychedelic harm reduction within event-health systems. The two-event field application was used to assess survey administration, completion, record linkage, item interpretability, respondent burden, and the operational relevance of selected measures. It was not designed or powered to determine effectiveness, estimate causal effects, or quantify reductions in workload, escalation, transport, or resource use.

### REDCap introduction

Surveys were administered using REDCap, a secure web-based research data-capture platform selected to support data security, research integrity, transparency, and scalability (55–58). This project used separate consent, pre-event, and post-event instruments, with branching logic and survey routing used to direct respondents to the appropriate questionnaire. Email-based record linkage allowed pre- and post-event responses to be associated when both were completed, while still allowing participants to complete only one questionnaire. Access controls, audit functions, and de-identification procedures were used to limit access to identifiable information and suppor**t** data integrity.

Importantly, REDCap includes rigorous safeguards such as audit trails, configurable user rights, field-level access controls, data export restrictions, and de-identification options to support data protection and integrity (59). Its organizational elements offer flexible project design, integrated forms and surveys, data dictionaries, reporting tools, export functions, codebook generation, and structured participant-level records, further supporting data collection, monitoring, and preparation for analysis (59). These advanced features bolster research transparency by strengthening the traceability and organization of the team workflow. By engineering role-based permissions, traceable data changes, standardized entry rules, error checks, and organized export procedures into a single system, REDCap strengthens accountability across the research process. For these reasons, REDCap was deemed an ethical, responsible, and strategic choice to support data collection, enhance research organization, and promote the secure handling of sensitive research information.

Using the REDCap system, the survey was separated into three instruments: consent, pre-event, and post-event. Because REDCap generates only one public survey link per project, all participants entered through a shared Consent instrument. Participants entered the survey via one of two links, which auto-populated a hidden field via URL parameter to indicate whether the participant was completing the pre- or post-event survey. A Survey Queue then routed the participant to the appropriate instrument after consent was submitted.

The collection of email addresses in the Consent instrument allowed pre- and post-event surveys to be associated and merged into one record. This design also accommodated participants who completed only one of the two questionnaires.

#### Setting and participants

The formative field application was conducted at two large-scale events in the Denver metropolitan area in spring 2026. Eligible participants were medical personnel employed by the contracted event-medicine provider and assigned to one of the participating events. Zendo Project personnel, attendees, security staff, event producers, ambulance personnel, and off-site medical providers were not surveyed. Because the total number of eligible or invited medical personnel was not formally recorded, a formal survey response rate could not be calculated.

#### Recruitment and survey administration

Survey links were distributed to eligible medical personnel before and after each event. The pre-event survey was available before service deployment, and the post-event survey was available following each event. Participation was voluntary, and individual survey responses were not shared with employers or supervisors. Duplicate, blank, and incomplete records were handled according to the retention rules described below.

#### Ethics and data protection

The BRANY SBER Institutional Review Board determined that this study was exempt from IRB review on April 8, 2026 (Protocol No. IRB00010793). Participants provided electronic informed consent before accessing the survey. Email addresses were collected solely for pre/post record linkage, stored using a secure encryption-at-rest system, and removed from the analytic dataset before analysis.

Survey domains and individual questionnaire items were developed iteratively by the multidisciplinary study team based on a review of the literature on mass-gathering health, psychedelic harm reduction, crisis response, implementation science, and event operations. Because no validated instrument existed to evaluate the feasibility and perceived operational relevance of psychedelic peer-support services in non-clinical event settings, survey items were designed to capture constructs identified by the investigators as central to implementation, including workload, psychological distress burden, interprofessional coordination, escalation, perceived safety, operational effectiveness, and attendee disposition. The resulting instrument was refined through multiple rounds of discussion among investigators with expertise in event medicine, psychedelic peer support, healthcare operations, engineering, public health, and research methodology prior to pilot deployment. The instrument had not undergone psychometric validation; the present application therefore assessed feasibility and item interpretability rather than reliability or construct validity.

### Measurements

This study develops a framework for the prospective evaluation of psychedelic harm reduction services and reports a formative application of selected medical-staff survey components. The broader framework identifies six interdependent stakeholder groups: event producers, police and security, festival health, on-site medical services, ambulance services, and off-site medical services. The present field application collected survey data only from on-site medical personnel. Accordingly, it assessed the feasibility of measuring preparedness, anticipated psychological-distress burden, perceived workload, safety concerns, coordination, escalation, and attendee disposition; it did not directly measure outcomes across all six stakeholder groups. Within this system, we seek to assess quantitative outcomes including the prevention of unnecessary hospitalizations, reductions in ambulance transports off-site, reductions in arrests and law-enforcement escalations, decreased reliance on on-site medical intervention for non-medical crises, and broader reductions in potential downstream measures for event producers through metrics such as insurance costs.

This stakeholder-based model reflects the actual operational environment in which psychedelic harm reduction services would function at an event, allowing different types of outcomes to be measured according to the kind of evidence each stakeholder group can provide. Within the broader framework, independently recorded outcomes could include arrests, ambulance transports, hospital referrals, medical workload, medication or restraint use, security contacts, and encounter disposition. These outcomes were not objectively measured in the present field application. Concrete operational outcomes such as arrests, ambulance rides, hospitalizations, and medical workload lend themselves to measurable metrics, while attitudes, perceptions, and assessments from security, medical teams, and other stakeholders are better captured through ordinal survey instruments such as Likert-type scales. This distinction is especially important in the context of a “legal gray area,” where respondents may be unfamiliar with these questions and may hesitate to answer in ways that could expose them to professional or legal risk. In that setting, relative and perception-based measures provide a practical and ethically appropriate way to capture stakeholder experience and help researchers develop the proper language in order to interact with each stakeholder group.

Event-based medicine presents unique challenges that have yet to be fully characterized by existing literature (60). Staff perceptions may provide useful information about perceived coordination, workload, safety, and implementation problems that are difficult to identify through administrative data alone. Such perceptions are not substitutes for objective operational outcomes but can inform the selection and wording of measures for prospective evaluation.

Likert-type items were used to measure sentiments held by participants. In the pre-event questionnaire, Likert items measured feelings of preparedness, anticipated impact of a peer support team on personal workload, and concern for team physical safety due to potential high-risk physical altercations or individuals requiring restraint. In the post-event questionnaire, Likert items measured perception of overall operational success, Zendo Project's impact on team concerns for personal physical safety, efficacy of Zendo Project's integration with medical and security teams, and Zendo Project's perceived impact on guests who had an encounter with emergency services while under significant distress. Because individual items could be skipped or rendered inapplicable through survey routing, denominators were calculated separately for each item. The number of retained survey records therefore differs from the number of responses available for some post-event outcomes.

## Clinical oversight and medical methodology

The sanctuary service utilizes a nested oversight model designed to mitigate risk, ensure real-time clinical observation, and interface seamlessly with event-level emergency medical services (EMS). This governance framework rests on five pillars: credentialed medical volunteers, rigorous screening, continuous on-site oversight, standardized escalation procedures, and a point-of-care electronic charting system overseen by the medical director for quality assurance.

### Medical triage by licensed professionals

The administration team verifies the active healthcare credentials of every medical triage volunteer to ensure current clinical expertise. Medical professionals may feel that it is acceptable to work in this space as it is a continuous space of service to others in need of professional medical screening. This credentialing was intended to support informed point-of-care observation and communication with event EMS during care transitions.

Within the Zendo model, credentialed healthcare professionals participate as trained peer-support volunteers operating under clearly defined organizational protocols rather than providing individualized medical care under the authority of their professional licenses, illustrating how professional expertise can ethically strengthen community-based support while preserving appropriate boundaries between peer support and clinical practice. The model is conceptually aligned with Good Samaritan principles of voluntary assistance and appropriate escalation, although implementation remains governed by site-specific organizational protocols and applicable legal frameworks. The legal and professional boundaries of this role vary by jurisdiction and event protocol and were not evaluated in the present study.

### Screening and intake governance

Participant entry is governed by a proprietary, standardized medical triage protocol approved by the medical director. As part of the overall intake process into the space, every individual undergoes a medical screening evaluating baseline physiological parameters, cognitive orientation, and potential contraindications or co-ingestions. This initial screen identifies individuals requiring immediate higher-level intervention by medical or crisis-response teams.

### On-site oversight and staffing architecture

To guarantee continuous safety, a medical triage volunteer is staffed at all times to provide direct accessibility and line-of-sight monitoring within the sanctuary space. Operational procedures dictate that the sanctuary must always sit in close proximity to a fully equipped first aid station capable of basic life support (BLS) with capacity to transfer to a higher level of care for advanced life support (ALS). When structurally embedded within a first aid station, oversight transitions to a collaborative, inter-service workflow between medical services and Zendo Project volunteers. In this configuration, an attending physician or lead paramedic assumes the supervisor position, anchoring a unified command structure that bridges the peer-support framework with event-level clinical governance.

### Adverse-event monitoring and escalation

Clear clinical triggers dictate the immediate escalation of a guest to higher-acuity services upon signs of physiological or psychological deterioration. Pre-event briefings between the medical director and adjacent service lines establish protocols for swift, unhesitating care transitions. During an escalation, peer-support sitters may accompany the guest to provide continuity of care, operating collaboratively with security or medical personnel while the sanctuary medical triage volunteer remains stationed at the intake point.

### Charting and quality assurance

Volunteers utilize a personal device-based electronic charting system for immediate documentation of intake, scheduled observation rounds, and departure assessments. The medical director reviews these charts in full during post-event debriefs to audit clinical diligence and validate the appropriateness of care within the space. This data-driven quality assurance loop directly informs operational iterations and volunteer training curriculum as the service scales.

### Analytical

In Welfare Economics, total social welfare, that is, the social value of an activity such as a music festival is the sum of the consumer surplus and producer surplus generated by that activity (61):

(1) Social Value = Consumer Surplus + Producer Surplus

Consumer surplus represents the value to attendees above the price of attending. For many festival attendees, the value of attending generally increases with the use of psychoactive substance (PAS):

A number of recent studies as well as more classic references associate themes such as *spiritual growth, transcendence, potentiating insight, getting in touch with one's inner world, potentiating creativity* as reported intentions behind PAS use in recreational settings, especially in outdoor environments such as rave and trance scenes (62).

At the same time, attendees understand that when using psychoactive substances in general, and psychedelics in particular, there is a small to moderate risk of undergoing a challenging experience. While approximately 39% of individuals who use classic psychedelics report having undergone at least one challenging or distressing experience in their lifetime (63), the probability of undergoing a challenging experience during any single festival attendance is substantially lower. One study of people who had suffered challenging experiences found that 30% of such experiences occurred in festival or rave settings (64). Given that only a minority of festival attendees use psychedelics on any given day, and most users do not experience a challenging experience per event, the overall incidence requiring intervention at a festival remains relatively low.

Challenging experiences may end with either good resolutions or bad resolutions, where good resolutions involve a guest receiving assessment and then being released back to the event, while bad resolutions involve adverse reactions and the potential need for off-site care. Although a challenging experience (C) provides substantially less value to users of psychedelic substances (P) than a nurturing experience (N),

(2) Consumer Surplus (P|N) >> Consumer Surplus (P|C),

challenging experiences that reach good resolutions (G) diminish the overall value to consumers far less than those that end in bad resolutions (B),

(3) Consumer Surplus (P|C, G) >> Consumer Surplus (P|C, B).

The expected consumer surplus for attendees who use psychedelics (P) is thus:

(4) E[Consumer Surplus(P)] = Pr(N) × CS(P|N)

+ Pr(C, G) × CS(P|C, G)

+ Pr(C, B) × CS(P|C, B),

Where E[Consumer Surplus′(P)] > 0

Pr(N) + Pr(C) = 1

Pr(N) > Pr(C)

Pr(C, G) + Pr(C, B) = Pr(C)

E: Expected Value

Pr: Probability

P: Psychedelic Substance

N: Nurturing Experience

C: Challenging Experience

G: Good Resolution

B: Bad Resolution.

Producer surplus represents the net benefit to organizers of hosting a music festival. It is calculated as total revenues minus total costs of production,

(5) Producer Surplus = Revenues – Costs.

Event organizers maximize producer surplus by maximizing the revenues from hosting events while minimizing the associated costs. Revenues include ticket sales and concessions. The relevant costs for the analysis at hand are those associated with attendee management. Such costs generally fall into three main categories: costs of security and crowd management, costs of on-site medical services, and costs of liability insurance. Together, these three categories typically account for 15–30% of the festival's total operations budget (65).

Consumption of psychedelic substances (P) by festival attendees increases operations costs across all three categories,

(6) Costs′(P) > 0.

While attendees who use psychedelic substances and have nurturing experiences generally do not increase operators' costs much, attendees facing challenging experiences often require elevated engagement from security and/or medical personnel. In rare but serious cases, such incidents can escalate, resulting in significant liabilities for event organizers. In short, the costs to festival hosts of managing events increase with use of psychedelic substances, with higher costs when attendees experience challenging experiences with good resolutions and much higher costs when attendees experience challenging experiences with bad resolutions,

(7) Costs(P|C, B) >> Costs(P|C, G) >> Costs(P|N)

Analogous to the expected consumer surplus for attendees who use psychedelic substances, the expected operations costs associated with attendees who use psychedelic substances are:

(8) E[Costs(P)] = Pr(N) × Costs(P|N)

+ Pr(C, G) × Costs(P|C, G)

+ Pr(C, B) × Costs(P|C, B).

If use of psychedelic substances increases prospective attendees' willingness to attend and/or willingness to pay for festivals by more than it raises costs for event organizers, then greater such consumption at these events increases overall social welfare.

In any case, given that the use of psychedelics is common among festival attendees exceeding 10% of attendees by some estimates (66, 67) and that a portion of users will face challenging experiences, both attendees and hosts benefit from interventions that raise the likelihood of positive resolutions [Pr(C, G)], reduce the likelihood of negative outcomes [Pr(C, B)] and decrease the respective costs, Costs(P|C, G) and Costs(P|C, B), from these experiences.

Within this conceptual framework, the Zendo Project seeks to increase the likelihood of favorable resolutions while derisking and reducing unnecessary escalation.

Zendo Project cannot decrease the probability that users of psychedelic substances face challenging experiences. However, Zendo Project is intended to increase the probability that festival attendees who do face challenging experiences end with good resolutions rather than bad ones. Zendo can also improve the nature of the experience, and thus the value to consumers of challenging experiences that end in good resolutions. Both factors increase the value of the event for existing attendees, while reducing operating costs for event hosts. In addition, the presence of Zendo Project may be likely to either attract new attendees who would otherwise not come, and/or lead more attendees to use psychedelic substances who otherwise would not, knowing that specialized support is available. One survey of festival attendees, most of whom planned to use drugs, suggests that the “presence of on-site medical services was a major factor when deciding to attend the festival” for a majority of attendees (68). Zendo Project would likely amplify this effect beyond standard on-site medical services. Since attendees who use psychedelic substances derive greater enjoyment from the festival experience while only a small proportion of such users face challenging experiences, the net effect of Zendo Project and similar support services is expected to be positive: the resulting increase in consumer demand may ultimately outweigh the modest rise in operating costs, generating a net increase in total social welfare.

### Data analysis

#### Quantitative analysis

Quantitative responses were summarized using counts and item-level response distributions. Because of the small sample, item nonresponse, and limited number of matched records, no inferential statistical tests or within-subject pre/post comparisons were conducted. Pre-event and post-event responses were treated as separate descriptive samples. Denominators are reported for each item. Event-level quantities estimated by respondents were treated as perceptions rather than independently verified encounter or utilization counts.

#### Qualitative analysis

Open-ended responses were analyzed using descriptive qualitative content analysis. Two members of the research team independently reviewed the responses and developed an initial coding framework based on the prespecified survey domains and recurring content. Coding differences were discussed and resolved by consensus. Because the qualitative dataset was small, the analysis was used to organize and contextualize responses rather than establish thematic saturation or population-level themes.

#### Mixed-methods integration

Quantitative and qualitative findings were integrated at the level of the prespecified evaluation domains. Open-ended responses were used to clarify how respondents understood ratings concerning workload, coordination, safety, and escalation. Integration was organized by linking each quantitative domain with corresponding qualitative observations and feasibility implications for prospective measurement.

### Technical: the serenity field application

To support systematic evaluation of field-based psychedelic harm reduction, Serenity was incrementally developed as a technical infrastructure for standardizing encounter documentation, improving data quality at the point of collection, and enabling responsible implementation in complex, emerging, trust-sensitive regulatory environments. By organizing time-stamped observations of intake, reassessment, intervention, and disposition, Serenity is also intended to support temporal abstraction, that is, the representation of changing encounter states as interpretable phases, trends, and transitions.

The development of Serenity, Zendo Project's internal data collection tool, was shaped as much by implementation realities as by technological considerations. Although ultimately realized as a software platform, Serenity originated as a paper-based system, and many of its core design principles emerged from the limitations and lessons of that earlier approach. Operating at events with more than 400 volunteers required reliance on individuals who often received a single training session and served only a small number of shifts. Long before software was introduced, substantial effort had already been devoted to optimizing paper workflows through color-coded forms, numbered records, dedicated binders, and standardized documentation procedures intended to reduce error and increase consistency. Despite these measures, records frequently contained incomplete information, contradictory timestamps, or other data quality issues. At the scale of large events, there was rarely a practical mechanism for identifying the source of documentation errors or correcting them retrospectively. These limitations highlighted an important principle: the point of data collection is often the only reliable opportunity to ensure data quality. This realization became a primary driver for the transition to software-supported workflows.

A key lesson from this process was that technology implementation in gray-market environments benefits from beginning at the lowest feasible level of complexity and risk. Rather than introducing a comprehensive digital system from the outset, the initial objective was to determine whether participants were comfortable with documentation itself. Paper-based workflows provided a low-risk mechanism for assessing acceptance, minimizing perceived surveillance concerns, and allowing quality assurance processes to be evaluated before introducing more sophisticated technologies. In this sense, paper functioned as both a documentation tool and a field-based feasibility assessment. Had participants rejected paper documentation, subsequent technological deployment would likely have required substantial redesign. Acceptance of paper documentation instead provided evidence that more advanced forms of data collection could be introduced incrementally.

The first software-based iteration, deployed in 2019, intentionally limited technological exposure by assigning a single iPad to each site. A designated logkeeper recorded guest arrivals and departures, reported substances, presenting concerns, sitter assignments, and bed locations. Importantly, technology was introduced through a single operational role rather than across the entire service environment. This phased approach reflected an implementation philosophy centered on participant and volunteer comfort rather than technological capability. Trust was established first with a single point of interaction before expanding functionality to additional roles. Sitters continued documenting on paper cards that were later photographed and attached to digital records, a compromise driven partly by resource constraints and partly by workflow realities. At shift change, multiple volunteers often needed to document simultaneously, making individual device allocation impractical (69). As trust, familiarity, and infrastructure matured, subsequent iterations expanded access to additional operational roles, including shift leads, medical triage personnel, and other coordinators, enabling multiple stakeholders to interact with shared data simultaneously.

This incremental progression revealed that innovation in emerging regulatory environments is often driven less by technological advancement itself than by the successful integration of technology with social, organizational, and regulatory realities (70). Effective implementation required continual navigation of hardware limitations, software capabilities, volunteer practices, operational culture, and evolving perceptions of risk. Rather than viewing innovation as a discrete technological event, the process functioned as a series of iterative adaptations that aligned technological functionality with the comfort levels and expectations of the individuals responsible for using the system. In this context, stakeholder trust and willingness to engage became primary indicators of implementation success.

Several design features emerged directly from these experiences. The application incorporates real-time validation during the guest departure process and prevents records from being finalized until required fields are completed or explanations are provided for missing information. As a result, data quality becomes an embedded property of the system rather than a responsibility placed solely on individual volunteers. This approach effectively transfers quality assurance from retrospective correction to prospective process design. Standardization that proved difficult to achieve through training alone became structurally encoded within the platform itself.

Another important design consideration involved the visibility of technology within care environments. Serenity was intentionally developed as what might be described as a *double-sided interface*, designed simultaneously for those entering information and for the individuals whose experiences were being documented. Many clinical information systems prioritize the needs of the documenter and assume data entry occurs directly in front of the patient. In contrast, Serenity was designed to minimize technological intrusion into supportive interactions. Sitters do not interact with devices while supporting guests. Documentation responsibilities remain with logkeepers, shift leads, and triage personnel, while sitter notes are completed after guest interactions conclude. Consequently, guests may spend their entire interaction without observing their sitter engage with a device. This design choice reflects an understanding that the visibility of technology can influence perceptions of safety, trust, and care quality, particularly in altered-state environments.

The same implementation philosophy informed system monitoring and evaluation. Traditional organizational development frequently relies upon active accountability mechanisms, such as individual performance tracking, ticketing systems, or fault attribution frameworks designed to identify responsibility for errors. While effective in many corporate settings, such approaches may create unintended barriers in gray-market environments where participants, volunteers, or developers may be concerned about documentation being used to assign blame or create future liability. Serenity therefore emphasized passive accountability mechanisms that focus on identifying process failures rather than individual failures. System logs are analyzed to identify workflow bottlenecks, training gaps, and operational inefficiencies without requiring volunteers to self-report problems or assume responsibility for them. For example, if arrivals are expected to be entered within fifteen min but system-wide data consistently demonstrates longer delays across multiple shifts and volunteers, this indicates a workflow or training issue rather than an individual performance issue. This approach preserves opportunities for continuous improvement while reducing concerns related to fault assignment and potential incrimination.

Importantly, the system itself was scaled according to the same principle. Expansion occurred from a single operational touchpoint outward rather than through organization-wide deployment. Initial constraints were largely technical, including networking limitations, internet connection intermittency at remote sites, and the inability of early software versions to synchronize across devices. However, these limitations ultimately reinforced a broader implementation strategy: introducing technology gradually allowed the organization to assess user acceptance, identify operational risks, and refine workflows before expanding functionality. The result was not merely a software platform, but an implementation model demonstrating how technology can be responsibly developed, validated, and deployed within emerging regulatory environments where trust, participant comfort, and risk mitigation may be as important as technological performance itself.

## Results

### Data

Pilot data were collected across two events in the Denver metropolitan area in spring 2026: a large-scale electronic music concert (estimated attendance 5,000–10,000) and a large-scale rock concert (estimated attendance 10,000–20,000+). The two events differed in genre, anticipated crowd behavior, and expected substance use profiles, providing variation in the operational context within which the Zendo Project's services were deployed.

### Sample

All respondents were medical staff employed by the contracted event-medicine provider at each event. No attendees, Zendo Project personnel, or security staff were surveyed. The sample therefore reflects the perspective of medical professionals with direct operational exposure to the peer support model.

The pre-event sample included 19 respondents. Among 18 respondents who answered the organizational-tenure item, 12 reported 1–5 years with their organization, 5 reported less than one year, and 1 reported 6–10 years. Five respondents identified as managers. Most respondents reported prior experience working alongside peer support, harm reduction or mental health staff in event settings: the most common response was 2–5 occasions (*n* = 10), followed by prior occasions (*n* = 5). Only one respondent reported no such prior experience.

### Survey-record disposition and completeness

Twenty-eight REDCap records were created across the two events. One record was excluded prior to analysis because the respondent declined consent. Of the remaining records, 19 completed pre-event surveys and 5 completed post-event surveys were retained for descriptive analysis. Table 1 summarizes the completed survey records included in the descriptive analysis by event and survey time point.

**Table 1:** Completed pre-event and post-event survey records by event.

Event	Pre-event completed	Post-event completed
Event 1 (electronic)	9	3
Event 2 (rock)	10	2
Total	19	5

The number of completed post-event surveys was substantially lower than the number of completed pre-event surveys, likely reflecting the logistical constraints of surveying on-duty medical staff after late event shifts. Five post-event surveys met retention criteria. One retained post-event record was incomplete for several principal operational-perception items, resulting in an item-level denominator of four for those findings. This record was retained because it contained other usable responses; the reduced denominator reflects item nonresponse rather than exclusion based on response content. Only two respondents completed both pre-event and post-event surveys. Meaningful within-subject comparison was therefore not feasible, and pre-event and post-event responses were treated as separate descriptive samples.

The field-application findings are presented as descriptive feasibility observations rather than estimates of intervention effectiveness. The application was used to assess survey administration, item completion, record linkage, item interpretability, and the relevance of proposed measurement domains under field conditions. The Denver pilot functioned as an internal, early-stage implementation opportunity to test survey domains, workflow feasibility, respondent burden, and the operational relevance of proposed measures before larger-scale data collection. Accordingly, the findings below are interpreted as directional signals used to refine the evaluation framework for subsequent deployments.

One retained post-event record did not contain responses to several principal operational-perception items; therefore, those post-event items are reported with an item-level denominator of four.

The pre-event responses were therefore used less as baseline measures for causal comparison and more as a field-informed assessment of expected operational pressures, perceived readiness, and the kinds of constructs that medical personnel considered relevant before service deployment. Pre-event responses indicated that medical personnel anticipated moderate operational demand. Most respondents expected 16–30 patient encounters (*n* = 9), followed by 6–15 encounters (*n* = 5), 31–50 encounters (*n* = 3), and 0–5 encounters (*n* = 2). Anticipated hospital transfers were most commonly estimated at 3–5 transfers (*n* = 15). Respondents generally described their teams as prepared for the expected event burden: 9 reported being “confidently prepared,” 6 “sufficiently prepared,” and 4 “completely prepared.” No respondent selected “not prepared” or “somewhat prepared.”

Respondents expected acute psychological distress to represent a meaningful portion of event-medicine workload. Nine respondents anticipated that 11–25% of encounters would involve acute psychological distress, five anticipated 26–50%, four anticipated 0–10%, and one anticipated more than 50%. Anticipated physically escalated individuals were most commonly estimated at 1–2 cases (*n* = 13), with smaller numbers expecting 3–5 cases (*n* = 3) or none (*n* = 2). Safety concerns were present but generally moderate: 10 respondents reported being “somewhat concerned,” three “concerned,” and six “not concerned.”

Pre-event expectations regarding the Zendo Project were generally favorable. Sixteen of 19 respondents expected the peer-support team to reduce workload, including 10 who expected a significant reduction and six who expected a slight reduction. One respondent anticipated no impact, while two anticipated a significant increase in workload. Open-ended pre-event responses described anticipated challenges related to crowd density, substance use, dehydration, fatigue, behavioral escalation, and the need for coordination across medical, security, and support personnel.

Four of the five retained post-event records contained responses to the principal operational-perception items. These item-level results are summarized descriptively and should not be interpreted as evidence of change from baseline or as representative of the full medical workforce.

Among the four respondents who completed the principal post-event operational-perception items, all reported that Zendo Project reduced strain on their team. The same four respondents completed the items concerning overall operations, estimated patient encounters, and hospital transfers; all four rated the overall medical/security operation as “extremely successful.” Their estimates of patient encounters ranged from 20 to 30, and each estimated six hospital transfers. These values represent respondent estimates of event-level activity and were not independently verified against incident or transport records. Two respondents estimated that 11–25% of encounters involved acute psychological distress, while two estimated that more than 50% did so.

Respondents also estimated the number of situations they believed may have escalated in the absence of Zendo support. Three estimated 6–10 such situations, while one estimated 1–2. These counterfactual estimates cannot establish how many escalations, transports, restraints, or sedations were prevented. Open-ended responses nevertheless indicated that respondents viewed extended observation, calming support, and the ability to redirect medical attention as operationally useful. One respondent described an incident they believed might otherwise have required sedation, and another described Zendo personnel remaining with distressed individuals while medical personnel attended to other calls.

In open-ended responses, staff described safe return to the venue, departure with a friend, and avoidance of transport as outcomes they associated with particular encounters. They also emphasized grounding, compassionate support, extended observation, and the availability of a lower-stimulation space. These accounts contextualize staff perceptions but do not independently establish attendee outcomes or transport avoidance.

Taken together, the responses indicated that participants could interpret and respond to items concerning psychological-distress burden, workload, integration, safety, escalation, time use, and attendee experience. The pilot also identified important implementation lessons, particularly the need for stronger post-event response capture, clearer procedures for linking pre- and post-event records, and expansion beyond managerial respondents to include a broader range of front-line medical, security, and event-health personnel. These refinements are being incorporated into the planned next phase of field data collection.

The small post-event sample, item nonresponse, and availability of only two matched pre/post records precluded meaningful within-subject or inferential analysis. The post-event responses should therefore be interpreted as descriptive staff perceptions rather than directional evidence of intervention effect. The empirical contribution of the field application is that the proposed survey domains produced interpretable responses while revealing important feasibility problems involving post-event recruitment, record linkage, item completeness, counterfactual wording, and reliance on estimated outcomes.

## Discussion

The present manuscript contributes to the emerging literature on psychedelic harm reduction by proposing a methodological framework and reporting a formative application of selected measurement components in two event settings. The field application examined whether medical-personnel surveys could generate interpretable information under live operational conditions. It did not determine how peer support affected attendee, medical, security, or resource-use outcomes. Much of the recent literature has focused either on conceptual harm-reduction models, clinical or psychotherapeutic frameworks, or retrospective accounts of adverse psychedelic experiences and their resolution (71–74). By contrast, this study examines how psychologically informed peer support functions when embedded within the operational realities of a live event, including uncertainty about substance use, rapidly changing environments, and the simultaneous presence of medical and security systems. In that sense, the study contributes ecologically valid evidence to a literature that still has relatively few evaluative models for non-clinical psychedelic support or experience.

Methodologically, one of the study's main strengths is that it makes field-based harm reduction more empirically legible. Rather than treating support encounters as informal or purely anecdotal, the design organizes them into structured domains such as presentation, triage, escalation, disposition, and interprofessional coordination. This matters because recent work has emphasized that psychedelic adverse effects remain underdefined and unevenly captured, particularly outside controlled settings (71, 73). Recent mixed-methods psychedelic research has also shown the value of translating qualitative accounts of difficult experiences into analyzable categories and measures (74). The present study extends that logic into a mass-gathering environment, where encounter documentation can clarify not only what kinds of distress occur, but also how they move through an event response system.

The study proposes and formatively applies an evaluation framework for describing field-based psychedelic support models. In the terms of this manuscript, the model is best understood not as a substitute for emergency or psychiatric care, but as a field-based response for medically stable guests whose primary needs are psychological containment, reassurance, and observation. Interpreted this way, the analytic focus on triage, escalation, and disposition is especially useful. These domains help clarify where peer support begins, where its limits lie, and when responsibility appropriately shifts to medical or public-safety teams. Even if finalized quantitative estimates of escalation and disposition are still pending, the study provides a methodology for examining how prospective studies might evaluate whether a peer-support model contributes to distinguishing lower-acuity altered-state distress from presentations that require higher-acuity intervention.

A feature of the field application is the inclusion of healthcare and security personnel feedback. Most psychedelic outcome studies center the individual user's retrospective account; that perspective is essential, but incomplete when the intervention is embedded within a multi-agency event system. Staff perceptions of workload, safety, integration, and encounter quality provide a complementary level of analysis that is operational rather than purely experiential. This aligns with broader crisis-response literature emphasizing that effective non-law-enforcement response models depend not only on de-escalation, but also on communication, relationship-building, role clarity, and safe handoff practices with adjacent services (11). In this respect, the study helps situate psychedelic peer support within the practical realities of coordinated event operations rather than treating it as an isolated interpersonal intervention.

More broadly, this framework suggests that future evaluations of psychedelic peer-support services may benefit from shifting emphasis beyond individual participant outcomes toward system-level measures such as interprofessional integration, operational coordination, resource utilization, and response pathways. Evaluating how peer-support services function within the broader event-health ecosystem may provide a more comprehensive understanding of their public health and operational value.

At the same time, the findings should be interpreted cautiously. As a case study of one established organization in naturalistic settings, the study is likely subject to limits common to field research, including event-to-event heterogeneity, observational rather than experimental inference, incomplete follow-up, and the possibility that staff perceptions are shaped by prior attitudes toward harm reduction. These constraints mean the study may be better read as an initial evaluative framework than as a definitive test of effectiveness. Nonetheless, this represents an important methodological contribution. As non-clinical psychedelic use continues to expand, research will need methods that can measure not only whether distress occurs, but how support systems document it, triage it, and coordinate around it in real-world environments.

### Policy implications

Psychedelic harm reduction at large-scale events may be understood as a bounded, complementary component of event health infrastructure rather than as a substitute for emergency medical services (EMS), security, law enforcement, or clinical care. The policy relevance of this distinction is strengthened by recent evidence showing that challenging psychoactive experiences are sufficiently common to be foreseeable among people who use psychedelics, while their severity, duration, and consequences are highly variable (63, 71, 73). In practical terms, psychoactive-related distress should not be treated solely as an exceptional, disciplinary or high-acuity medical problem. Instead, it should be approached as a predictable public health presentation requiring advance planning, defined triage pathways, and coordinated response protocols.

From a policy perspective, the Zendo Project model is most defensible when positioned in the intermediate zone between self-limited distress and presentations that clearly require medical or public safety intervention. A trained psychoactive substance harm reduction team can serve a specific systems role: identifying medically stable but psychologically distressed guests, providing supportive containment in a lower-stimulation environment, and escalating promptly when medical or safety red flags emerge. This framing aligns with recent mass-gathering health literature emphasizing psychosocial interventions, context-sensitive support, and the need for structured health planning at large events (10, 68). It also avoids overstating the role of peer support: Zendo-type services are not alternatives to EMS or security, but rather complementary pathways that may help match the level of response to the nature of the presentation.

This distinction is especially important because the policy significance of a difficult psychoactive experience depends not only on whether distress occurs, however also on how that distress resolves. Therefore, difficult psychoactive experiences may have different individual and system-level consequences depending on whether they resolve safely or escalate into medical, security, or operational concerns. This study conceptualizes these relationships using consumer and producer surplus, including the potential effects of difficult experiences on attendee value and organizer costs related to medical services, security, crowd management, and liability risk. In policy language, this supports a narrower and more defensible claim: event systems should be designed to support safer resolution, improve triage, de-risk stakeholders, and reduce avoidable escalation where possible. Claims about cost savings, reduced EMS burden, or liability reduction should remain conditional until supported by finalized outcome data.

The proposed Zendo Project triage model can help operationalize this policy by depicting peer support as one defined pathway within an integrated event-health system. In this model, a guest in distress first enters an initial safety and medical screen. Medical red flag routes the guest to EMS or on-site medical care, while imminent risk to self or others routes the guest to security or public safety. When neither threshold is met, the guest may be routed to trained Zendo Project peer support for monitoring, deescalation, reassurance, and environmental containment. Importantly, the model should depict triage as dynamic: guests initially supported through peer care may later require escalation if risk status changes. Capturing these reassessments over time permits temporal abstraction of the care pathway, allowing researchers to distinguish stable presentations, improvement, deterioration, and transitions to higher-acuity services.

Documentation is a second major policy implication. Psychedelic harm reduction services are easier to integrate into formal event planning when they produce auditable, de-identified records that clarify presentation type, interventions delivered, disposition, and reasons for escalation. Standardized documentation can support quality improvement, improve handoff quality when guests are transferred to medical teams, and generate event-level data to inform staffing, training, and future risk planning. This is particularly important given recent calls for more systematic adverse-event monitoring and reporting in psychedelic research and practice (73, 75). For public health systems, documentation is not merely administrative; it is what makes field-based care evaluable, scalable, and accountable. As regulatory environments mature, policymakers should remain attentive to the possibility that increasing institutionalization may inadvertently create forms of *legitimized complexity*, in which additional procedural, administrative, or clinical layers become normalized despite offering limited incremental benefit relative to lower-cost community-based interventions (76).

Overall, the policy case for the Zendo Project model is not that peer support replaces professional care, but that it may occupy a currently under-specified operational space within mass-gathering health. Large-scale events already plan for dehydration, injury, intoxication, and crowd risk; foreseeable psychedelic-related psychological distress should be addressed with similar systems logic. Policies that formalize triage thresholds, peer-support scope of practice, documentation requirements, medical oversight, and handoff rules with EMS and security are likely to be more scalable and defensible than *ad hoc* or punitive responses. In this sense, the Zendo Project model can be understood as a pragmatic event-health innovation: a structured layer of care for a predictable category of distress that existing systems are not specifically designed to address.

### Clinical implications

Zendo Project's low-barrier, non-clinical, trauma-informed crisis support model exists within a broader continuum of crisis care at the intersection of medical and psychological fields. It occupies a distinct layer between no support and medical intervention, applicable across altered states presenting in the event context including psychedelic, poly-substance, grief, and acute anxiety. When embedded alongside medical services, the model is designed to provide an intermediate pathway for medically stable guests while preserving escalation routes for clinical or safety concerns. Whether this arrangement improves attendee outcomes or reduces personnel burden requires prospective evaluation.

Prior studies of peer-based psychedelic support and adjacent crisis-response models provide preliminary reasons to evaluate whether such services may affect emergency-service use (28, 62, 77, 78). However, the present field application did not independently measure emergency-department contacts, psychiatric-service use, law-enforcement contact, or transport avoidance. Prospective research should evaluate these outcomes using linked incident records and appropriate comparison conditions.

Zendo Project's approach is grounded in empirically derived principles of acute psychological care. Practices that promote a sense of safety, calming, self- and community efficacy, connectedness, and hope are identified as key components of effective acute field-based psychological support (79). Zendo Project's non-clinical volunteers provide trauma-informed, non-coercive approaches to crisis care, recognized as best practice in crisis care settings, and especially so in acute crisis settings (80), and are intended to minimize unnecessary escalation and the risk of retraumatization.

Zendo Project's event-based setting introduces clinical complexity not fully addressed by existing field-based crisis frameworks. Psychedelics can evoke or compound unprocessed recent or historical trauma, producing nervous system activation and distress consistent with acute trauma response. Trauma surfacing during psychedelic states may manifest as direct trauma re-experiencing, symbolic/somatic re-embodiment, and fragmentation and confusion (81). At times, symptoms of trauma-induced or substance-induced states may be indistinguishable from medical emergency, which exceeds the role and training of volunteers, thus necessitating close coordination with medical teams for rapid assessment and treatment as necessary.

A central clinical implication of the Zendo Project model is that psychological distress and medical emergency must be distinguished without prematurely assuming either. In psychedelic and other altered-state presentations, panic, dissociation, trauma activation, confusion, somatic fear, and fear of dying may appear as hyperventilation, shaking, chest tightness, agitation, disorientation, or impaired communication; such features can reflect severe psychological distress without, by themselves, establishing medical instability, and trauma-informed containment may be appropriate when initial screening indicates stability (74, 80). At the same time, challenging psychedelic experiences can include physical distress, paranoia, dissociation, and trauma-related re-experiencing or fragmentation, which may resemble intoxication syndromes, evolving medical instability, or emergent safety risk (74, 81). Zendo Project-style care should therefore be framed not as a substitute for clinical assessment, EMS, or medical treatment, but as a complementary layer for signal distillation operating within clear screening procedures, red-flag thresholds, medical coordination, and repeated reassessment. Clinically, the model's value is response-matching: psychological containment when appropriate, rapid escalation when necessary.

### ASCI

Many of the principles and practices embodied within the Zendo Project can be interpreted through the Theory of Altered State Compassionate Integration (ASCI), a conceptual framework positing that empathic, emotionally attuned support during transitions into or out of altered states facilitates psychological coherence and mitigates trauma by reinforcing trust, safety, and meaning-making processes that transactional or procedural approaches may not reliably activate (82). ASCI is advanced here not as a validated instrument, empirical assessment scale, or established causal model, but as a theoretical lens: a shared vocabulary and set of organizing constructs for describing what compassionate presence may be doing at psychological, relational, and integration levels during altered states. These observations are consistent with broader psychedelic research showing that psychedelic states involve marked changes in brain dynamics (83) and that outcomes are shaped by set and setting, that difficult psychedelic experiences may carry both acute risks and potential long-term benefits, and that structured support can influence how such experiences unfold before, during, and after their occurrence (72, 74, 84, 85). ASCI also draws on broader evidence that relational factors, including therapeutic alliance, empathic communication, perceived support, and meaning-making, are associated with psychological adjustment and clinical outcomes across helping contexts (86–88). The present study examines ASCI principles in practice, illustrating how compassionate peer-driven interventions complement, and at times fill critical gaps in, the security and medical architecture of large-scale events, and offering an initial methodology for characterizing their value.

The value generated by Zendo Project's work, observable at the participant, provider, and system levels, points to a form of under-recognized value that ASCI is designed to illuminate. This pattern may be particularly common among organizations that operate in a pre-profit capacity, investing in the infrastructure, knowledge, and public goods necessary for emerging markets to mature before sustainable commercial models can develop. Standard cost-accounting and health-care utilization models more readily capture discrete, billable, and institutionally legible events, such as ambulance transport, emergency department use, hospitalization, or pharmacological intervention, than they capture the relational, deescalatory, and functionally preventative labor of peer-driven compassionate support offered to an individual navigating a difficult psychedelic experience (89, 90). This asymmetry matters because event-based harm-reduction models suggest that non-medical support services can operate alongside medical services as complementary public health infrastructure, informing triage, reducing acuity, and potentially decreasing unnecessary use of higher-cost medical, emergency, or security interventions (22, 28).

Because this middle layer of psychological and relational intervention resists conventional resource-use measurement, it may be under-recognized and consequently under-prioritized in resource allocation and care-pathway design relative to discrete, billable, and institutionally legible services (89, 91–93). ASCI names this measurement bias and provides a conceptual scaffold for recognizing the functional, rather than ornamental, role of compassionate presence in stabilizing both individuals and the systems through which they move.

A further challenge arises from the fact that some of the mechanisms ASCI seeks to describe may derive their effectiveness precisely from being difficult to commodify. Conventional economic systems are generally better equipped to recognize and exchange value through transactional relationships, often characterized by formal roles, asymmetries of authority, and clearly defined services, than through relational or interpersonal forms of care whose value is often under-recognized or omitted from conventional economic accounting within such frameworks (89, 92). In contrast, peer-based support is defined less by the delivery of a discrete service than by the relational position from which support is offered. Rather than an expert acting hierarchically upon a recipient, the supporter is positioned as a fellow participant whose credibility derives from presence, attunement, experiential understanding, and shared humanity. Peer-support literature suggests that this kind of relational positioning can foster mutuality, empowerment, social connection, and trust in ways that distinguish peer support from more formal provider–recipient relationships (94–96).

This distinction is not meant to suggest peer support as a substitute for professional care. Medical emergencies, psychiatric risk, intoxication syndromes, and cases requiring clinical assessment demand appropriately trained responders. Rather, the relational conditions described above help explain peer support's complementary value: it can occupy a functional and psychological gap between no intervention and higher-acuity response. By establishing trust, reducing perceived threat, and supporting communication, compassionate peer support may both reduce unnecessary escalation and make higher-acuity services more effective when escalation is required, helping a distressed individual become more reachable, communicative, and responsive to assessment, handoff, or transport (22, 28). In this role, peer support generates value that is systemically important but difficult to price, exchange, or evaluate within frameworks organized around observable transactions.

The same feature that makes compassionate peer support difficult to commodify at the systems level may also help explain its psychological force at the participant level: it is experienced as care outside the logic of transaction. Unlike many social interactions structured around exchange, evaluation, authority, or obligation, compassionate peer support may create a temporary relational space in which individuals are not required to perform, justify, negotiate, or earn care. This suspension of transactional expectations may be particularly important during periods of psychological vulnerability, allowing trust, agency, and emotional safety to emerge in ways that facilitate integration and reduce the likelihood of distress becoming further amplified or internalized. This proposed mechanism is consistent with evidence linking alliance, empathic communication, social support, and meaning-making with psychological adjustment and improved outcomes across clinical and post-traumatic contexts (86–88).

From this perspective, the difficulty of measuring the value of compassionate peer support may not simply reflect a lack of appropriate existing metrics, but a deeper mismatch between systems designed to quantify vertically exchanged services and forms of value that emerge through relational, reciprocal, and non-transactional human connection. This dynamic reflects how competitive markets tend toward conditions in which measurable and exchangeable forms of value are efficiently incorporated into price, while benefits arising from compassion, trust, social connection, and relational forms of care frequently remain externalized or under-recognized (89, 92).

Framing the value of Zendo Project's work in these terms and in this broader context is a necessary first step toward research, policy, and economic models that can account for compassionate care as infrastructure rather than amenity. While the present manuscript focuses on psychedelic harm reduction at large-scale events, the dynamics that ASCI describes, entry into and exit from altered states, the dependence of outcomes on relational safety, and the structural under-measurement of compassionate labor, are not decidedly unique to this context. They may extend to a broader class of domains in which individuals routinely operate at the edge of ordinary cognitive and emotional functioning. Future research should test these propositions directly, distinguish ASCI's proposed mechanisms from adjacent constructs, and develop methods capable of capturing both immediate de-escalation and downstream system-level value.

### Generalizability and future extensions

The present field application does not test ASCI, altered-state integration, or the generalizability of the framework beyond psychedelic harm reduction in event settings. ASCI is included here as a prospective, hypothesis-generating construct that may inform future measurement of relational support, psychological safety, meaning-making, and compassionate presence during altered-state distress. Any application of ASCI beyond psychedelic event settings, including to other high-stress or high-performance contexts, requires independent empirical study. Accordingly, the present manuscript treats ASCI as a future research direction rather than as an empirically supported conclusion from the current field application.

## Limitations

Limitations constrain the interpretation of this pilot study. First, the sample was small and drawn from two events served by a single event-medicine provider in one geographic region, limiting generalizability to other venues, event types, service models, and jurisdictions. Second, the post-event analytic sample was small, and only two respondents completed both pre-event and post-event surveys. As a result, paired pre-post analysis was not feasible, and findings should be interpreted as independent, descriptive observations rather than evidence of statistical change.

Third, the sample included medical personnel only. Attendees, Zendo Project volunteers, security staff, event producers, ambulance services, and off-site medical providers were not surveyed, meaning the findings reflect one operational perspective within a broader event-health system. Fourth, several outcomes, including perceived workload reduction, time saved, and prevention of escalation or transport, were based on staff perception rather than independently verified incident data. Finally, the study was observational and lacked a comparison condition, so causal claims about the Zendo Project's effectiveness cannot be made.

The low level of post-event completion creates a substantial risk of nonresponse and positive-response bias, because personnel with particularly favorable or salient experiences may have been more likely to complete the post-event survey. The investigator-developed instrument had not undergone psychometric validation, and the field application therefore assesses feasibility and interpretability rather than reliability or construct validity. Several respondents had prior experience with peer-support or harm reduction services, which may also have influenced expectations and ratings.

In addition, multiple respondents estimated the same event-level outcomes, and these estimates should not be treated as independent observations of encounters, transports, or escalations. Item-level missingness further reduced the available denominator for several findings. The field application did not link staff perceptions to independently verified encounter, transport, medication, restraint, staffing, or security data.

These limitations are consistent with the study's purpose as an initial feasibility and methodology paper. The data are best understood as illustrating that the proposed framework can generate operationally relevant field observations, while identifying refinements needed for larger, multi-site evaluations.

## Future research

This study represents an initial effort to develop a methodological framework for evaluating psychedelic harm reduction systems operating within non-clinical environments. Future research should focus on validating and refining this framework across diverse event types, regulatory regimes, populations, jurisdictions, and operational models. Future studies should also evaluate the reliability, validity, and reproducibility of the proposed measurement framework across different event types, organizations, and operational settings. Comparative studies examining multiple peer-support organizations, event environments, and response systems would help determine which factors most strongly influence safety outcomes, resource utilization, participant experiences, and organizational effectiveness. Prospective validation should distinguish clearly between operational outcomes, resource-use outcomes, and ASCI-related relational constructs.

An important next step will be the development of standardized outcome measures capable of assessing the public health, operational, and economic impacts of non-clinical support systems. While existing research has largely focused on individual experiences, future work should expand to include system-level outcomes such as emergency medical utilization, security engagement, workforce wellbeing, escalation rates, incident resolution pathways, and broader measures of event resilience. Longitudinal research may also help determine whether the effects of supportive interventions extend beyond the immediate event context and influence subsequent help-seeking behaviors, psychological integration, or community engagement.

As psychedelic use expands beyond clinical settings, future studies should examine how non-clinical support systems interact with healthcare providers, public safety organizations, insurers, venue operators, and policymakers. Particular attention should be given to identifying the conditions under which intermediate care pathways may improve coordination between existing services while reducing avoidable escalation to higher-acuity interventions. Such work may contribute to the development of evidence-based standards, best practices, and policy frameworks capable of supporting the safe integration of psychedelic-related services into broader public health infrastructure. Such work may also inform the development of quality assurance and evaluation frameworks for emerging psychedelic products, services, training programs, and digital care platforms, helping establish consistent standards for safety, effectiveness, implementation fidelity, and operational accountability across diverse settings.

Beyond psychedelics, the framework proposed here may have relevance for a wider range of altered-state, crisis-response, and psychologically intensive environments. Future research should explore its applicability to settings involving grief, trauma, disaster response, emergency services, wealth disparity, racism, increasing use of artificial intelligence, military and veteran populations, corporate worklife and management of teams, behavioral health crises, and other contexts in which outcomes may be influenced by the availability of compassionate, psychologically informed support.

## Conclusion

This study proposes a methodological framework and reports a formative field application used to refine selected measurement components. The two-event observations do not establish that the Zendo Project reduces workload, prevents escalation, improves attendee outcomes, or generates resource or economic savings. They demonstrate that medical personnel could provide interpretable feedback across several proposed domains while revealing substantial challenges involving recruitment, missingness, record linkage, and objective outcome verification.

The framework's economic and ASCI components remain prospective and hypothesis-generating. Their inclusion is intended to make the broader research program testable, not to generalize from the present sample. The next phase requires prospective, multi-site evaluation using predefined outcomes, linked operational records, broader stakeholder participation, validated measures, and appropriate comparison conditions. As new service models, technologies, training programs, and care infrastructures emerge, the ability to evaluate their effectiveness using transparent and evidence-based frameworks may become increasingly important to researchers, practitioners, innovators, developers, policymakers, regulators, and courts seeking to understand what constitutes safe, effective, and responsible care in rapidly evolving environments.

## Data Availability

The aggregated data supporting the conclusions of this article are included in the article. Participant-level data are not publicly available because the small sample and event-specific context may create a risk of participant identification. Further inquiries can be directed to the corresponding author.
